# [^68^Ga]Ga-PSMA-11 PET/CT in medullary thyroid carcinoma: comparison with [^18^F]FDG PET/CT and immunohistochemical analysis

**DOI:** 10.3389/fendo.2025.1627500

**Published:** 2025-10-01

**Authors:** Klaudia Zajkowska, Elwira Bakuła-Zalewska, Paulina Cegla, Marta Wojewódzka-Mirocha, Paweł Ochman, Agata Sackiewicz, Joanna Januszkiewicz-Caulier, Joanna Długosińska, Małgorzata Czetwertyńska, Marek Dedecjus

**Affiliations:** ^1^ Department of Endocrine Oncology and Nuclear Medicine, Maria Skłodowska-Curie National Research Institute of Oncology, Warsaw, Poland; ^2^ Department of Pathology and Laboratory Diagnostics, Maria Skłodowska-Curie National Research Institute of Oncology, Warsaw, Poland

**Keywords:** medullary thyroid carcinoma, PSMA, FDG, PET/CT, calcitonin, immunohistochemistry

## Abstract

**Introduction:**

Medullary thyroid carcinoma (MTC) is a rare neuroendocrine malignancy. Despite the use of various imaging modalities, including positron emission tomography combined with computed tomography (PET/CT), a significant proportion of patients with biochemical evidence of disease have no detectable lesions. Prostate-specific membrane antigen (PSMA) is expressed by the neovasculature of several solid tumours, including thyroid cancer. While case reports suggest that PSMA-targeted PET/CT may detect MTC lesions, its diagnostic value remains unverified. This study aimed to compare the clinical utility of [^68^Ga]Ga-PSMA-11 PET/CT with that of 2-[^18^F]fluoro-2-deoxy-D-glucose ([^18^F]FDG) PET/CT in MTC patients, assess PSMA expression by immunohistochemistry, and correlate PSMA expression with [^68^Ga]Ga-PSMA-11 PET/CT findings.

**Methods:**

Twelve patients with MTC who had undergone total thyroidectomy and presented with elevated serum calcitonin and/or carcinoembryonic antigen levels underwent prospective evaluation with [^68^Ga]Ga-PSMA-11 and [^18^F]FDG PET/CT. Immunohistochemical staining for PSMA and CD31 was performed.

**Results:**

The detection rate by [^68^Ga]Ga-PSMA-11 PET/CT was 100% (8/8) for lesion-based analysis, and 36.4% (4/11) for patient-based analysis, whereas those for [^18^F]FDG PET/CT were 12.5% (1/8) and 9.1% (1/11), respectively. [^68^Ga]Ga-PSMA-11 PET/CT led to a change in the clinical management of one (8.3%) patient. TBR_Blood_, and TBR_Liver_ were significantly higher on [^68^Ga]Ga-PSMA-11 PET/CT than on [^18^F]FDG PET/CT (p = 0.018 and p = 0.038, respectively). Immunohistochemistry confirmed neovascular expression of PSMA in 55% of patients. Expression was significantly higher in patients with positive [^68^Ga]Ga-PSMA-11 PET/CT results (p = 0.042).

**Conclusions:**

[^68^Ga]Ga-PSMA-11 PET/CT demonstrated higher detection rates than [^18^F]FDG PET/CT in both lesion-based and patient-based analyses.

## 1 Introduction

Medullary thyroid carcinoma (MTC), which originates from the parafollicular cells of the thyroid, accounts for approximately 2% of all thyroid malignancies (1, 2). As a neuroendocrine tumour, MTC has the capacity to secrete peptides such as calcitonin and carcinoembryonic antigen (CEA), which serve as serum biochemical markers for this tumour (3). MTC occurs in two forms: sporadic, accounting for approximately 75% of cases, and hereditary, accounting for 25% of cases. The hereditary form is a component of multiple endocrine neoplasia type 2 (MEN2), an autosomal dominant tumour syndrome caused by germline mutations in the REarranged during Transfection (RET) proto-oncogene (4). Somatic mutations in the RET proto-oncogene are detected in around 60% of sporadic MTC cases (5–7), and are associated with worse clinical outcomes (8).

Over the past two decades, there has been a consistent increase in both the incidence and mortality of MTC (9). Approximately half of patients present with advanced-stage disease (stages III–IV) at diagnosis (10), with no significant trend toward earlier-stage detection observed (11, 12). Although MTC accounts for only 2.2% of thyroid cancers, it is more aggressive than the more common follicular cell–derived well-differentiated thyroid cancers, making it responsible for up to 8% of all thyroid cancer-related deaths (2).

The treatment of choice is surgical management, typically involving total thyroidectomy with risk-adapted neck dissections (13–15). Measurement of calcitonin and CEA levels is recommended 2–3 months after surgery, and later during follow up (13, 14). Postoperative normalization of calcitonin levels is associated with a favourable outcome, while persistently elevated or rising calcitonin levels indicate persistent or recurrent disease, which warrants further diagnostic evaluation (13, 16, 17). According to current guidelines, patients with postoperative calcitonin levels < 150 pg/ml should undergo a physical examination and neck ultrasound, as these levels are typically indicative of locoregional disease. Conversely, calcitonin levels > 150 pg/ml are more frequently associated with distant metastases, and such patients should be evaluated using imaging modalities such as neck ultrasound, chest computed tomography (CT), contrast-enhanced magnetic resonance imaging (MRI) or three-phase contrast-enhanced CT of the liver, bone scintigraphy, and MRI of the pelvis and axial skeleton (13). Positron emission tomography combined with computed tomography (PET/CT) is usually recommended after previous negative cross-sectional anatomic studies (18). Several PET radiopharmaceuticals are used to image MTC, including 2-[^18^F]fluoro-2-deoxy-D-glucose ([^18^F]FDG), [^18^F]dihydroxyphenylalanine ([^18^F]FDOPA) and somatostatin analogues labelled with ^68^Ga ([^68^Ga]Ga-SSA); however, recommendations from scientific societies regarding their use are conflicting (13–15, 18). Despite the wide range of available imaging modalities, pathological lesions remain undetectable in approximately 20–35% of patients, even those with very high calcitonin levels (19, 20).

Prostate-specific membrane antigen (PSMA) is a transmembrane glycoprotein encoded by the folate hydrolase 1 (FOLH1) gene, initially identified on prostate cancer cells (21). Despite its name, PSMA is not prostate specific; its expression has been observed on the endothelium of tumour-associated neovasculature in various solid tumours, including thyroid cancer (22, 23). Preclinical studies suggest that PSMA plays a role in tumour angiogenesis by regulating integrin activation and signal transduction in a laminin-specific manner (24–26).

High expression of PSMA by prostate cancer cells is utilized for both diagnosis and treatment of prostate cancer. PSMA imaging is performed using PET/CT after administration of radiolabelled PSMA ligands such as [^68^Ga]Ga-PSMA-11, [^18^F]PSMA-1007 or [^18^F]DCFPyL, whereas PSMA-targeted PET/CT is used for initial staging, localization of recurrence, detection of prostate cancer classified as non-metastatic on conventional imaging, and evaluation prior to PSMA-directed radioligand therapy (27). Given that PSMA is expressed by the neovasculature of various solid tumours, PSMA PET/CT is also being investigated in the context of non-prostate cancers, including primary brain tumours, breast cancer, lung cancer, and renal cell carcinoma (28). The utility of PSMA PET/CT for imaging various histological types of thyroid cancer, including differentiated thyroid carcinoma, poorly differentiated thyroid carcinoma, and anaplastic thyroid carcinoma, has been demonstrated (29–32). Studies suggest that this modality not only identifies metastatic lesions across different locations and tissues, but also detects lesions that are undetectable by other nuclear medicine imaging techniques such as widely used iodine-131 whole-body scan and [^18^F]FDG PET/CT (29–32). To date, no studies have evaluated the utility of PSMA PET/CT in patients with MTC. Available data are limited to case reports confirming uptake of radiolabelled PSMA ligands by primary tumours and metastatic lesions of MTC (33–37). Immunohistochemical studies demonstrate expression of PSMA by the vasculature in up to 90% of patients with MTC (23, 38), suggesting that PSMA-targeted PET/CT could serve as a valuable diagnostic tool for this patient population.

The aim of this study was to evaluate the utility [^68^Ga]Ga-PSMA-11 PET/CT in MTC patients with elevated tumour markers and previously known lesions, as well as in those without detectable lesions on conventional imaging studies. A secondary objective was to assess the immunohistochemical expression of PSMA in tumour-associated vasculature, and its correlation with [^68^Ga]Ga-PSMA-11 PET/CT findings.

## 2 Methods

### 2.1 Patient selection

This was a single-centre, prospective study approved by the local bioethics committee (approval no. 28/2022, 04/12/2022) and conducted from 07/01/2022 to 03/31/2024. All patients provided written informed consent.

Adult patients with a histopathologically-confirmed diagnosis of MTC, all of whom had undergone total thyroidectomy and presented with elevated calcitonin levels suggestive of persistent or recurrent disease, were included. Exclusion criteria were as follows: adverse reactions to any of the radiopharmaceuticals used in the study ([^68^Ga]Ga-PSMA-11 or [^18^F]FDG); pregnancy or breastfeeding; a history of malignancy other than MTC; ongoing anticancer therapy during the study period; and an expected survival time of <3 months. Eligible patients were divided into two groups: those with known metastatic lesions and those without detectable lesions on conventional imaging studies (neck ultrasound, contrast-enhanced CT of the neck, chest, abdomen, and pelvis, and dynamic contrast-enhanced liver MRI), which were performed for all patients prior to study enrolment. Patients underwent prospective evaluation with [^68^Ga]Ga-PSMA-11 PET/CT and [^18^F]FDG PET/CT at an interval of approximately 100 days (± 30 days).

### 2.2 [^68^Ga]Ga-PSMA-11 PET/CT

[^68^Ga]Ga-PSMA-11 was prepared in accordance with Good Manufacturing Practice. Ready-to-use kits containing 20 µg of PSMA-11, with sodium acetate as a buffering agent (POLATOM, Owock, Poland) were used. Radiolabelling was performed using a GalliaPharm^® 68^Ge/^68^Ga Generator (Eckert & Ziegler, Berlin, Germany). The radiolabelling process was carried out at 95 °C for 10 min, followed by cooling at room temperature for an additional 10 min. Quality control of the generated [^68^Ga]Ga-PSMA-11 was conducted using thin-layer chromatography (TLC) with radiometric detection. The quality control process employed a developing solution comprising 1 mol/dm^3^ ammonium acetate and methanol in a 1:1 (v/v) ratio, along with ITLC-SG strips. The radiochemical purity of the [^68^Ga]Ga-PSMA-11 used for diagnostic purposes was no less than 97%.

[^68^Ga]Ga-PSMA-11, with an activity of 2 MBq/kg (0.054 mCi/kg), was administered intravenously approximately 60 min (range, 60–68) prior to PET/CT acquisition. A PET/CT scan without contrast was performed from the skull apex to the upper thighs using a Philips Gemini TF 16 scanner (Philips Healthcare, Cleveland, OH, USA). The imaging protocol involved a 3-min PET acquisition per bed position and low-dose CT (up to 100 mA, 120 kV), with a slice thickness of 3 mm and ordered-subsets expectation-maximization (OSEM) reconstruction.

### 2.3 [^18^F]FDG PET/CT

[^18^F]FDG was delivered to the Department by an external manufacturer (Synektik, Warsaw, Poland), who ensures the quality control of the radiopharmaceutical in accordance with the European Pharmacopoeia.

Patients fasted for at least 6 h prior to the examination. Serum glucose levels were measured prior to administration of [^18^F]FDG. Glucose levels did not exceed 150 mg/dL in non-diabetic patients and 200 mg/dL in diabetic patients. A PET/CT scan (Philips Gemini TF 16 scanner; Philips Healthcare, Cleveland, OH, USA) without contrast was performed from the skull apex to the upper thighs approximately 60 min (range, 60–67) after intravenous administration of [^18^F]FDG at an activity of 3.7 MBq/kg (0.1 mCi/kg). The imaging protocol involved a 3-min PET acquisition per bed position and low-dose CT (up to 100 mA, 120 kV), with a slice thickness of 3 mm and OSEM reconstruction.

### 2.4 Image analysis

PET/CT image analysis using the MIM workstation, version 7.3.5 (MIM Software Inc., Cleveland, OH, USA) was performed by two nuclear medicine physicians and one radiologist, with the final results agreed through consensus. The analysis included attenuation-corrected PET images and PET/CT images. During visual assessment, lesions demonstrating non-physiological uptake of the radiotracer that was significantly higher than that by surrounding tissues were classified as positive.

Semiquantitative analysis was performed using standardized uptake values (SUVs), normalized by body weight, and tumour-to-background ratios (TBRs). For positive lesions, the maximum and mean SUVs (SUV_max_ and SUV_mean_) were calculated with the PETEdge+ segmentation toll, which automatically generates volumes of interest (VOIs) based on an active contour algorithm. This algorithm identifies areas of elevated voxel intensity, and then uses spiral derivatives to refine lesions boundaries. If a lesion was positive by only one modality (either [^68^Ga]Ga-PSMA-11 or [^18^F]FDG PET/CT), the VOI was manually positioned at the corresponding anatomical location on CT to ensure consistent measurement. SUVs were also measured for a 3-cm diameter spherical VOI that was placed manually in the unaffected right upper lobe of the liver, and a 1-cm diameter spherical VOI placed in the aortic arch; these were used as reference tissues for calculation of TBR_Liver_ and TBR_Blood_ (TBR = lesion SUV_max_/reference tissue SUV_mean_).

### 2.5 Immunohistochemical staining and analysis of PSMA and CD31

Immunohistochemical staining was performed on formalin-fixed paraffin-embedded (FFPE) tissue blocks. In most patients, the material was obtained from primary tumours, while in one case staining was performed on a cervical lymphadenectomy specimen resected approximately one year after the initial surgery. The most representative and well-preserved tumour areas were selected. Immunohistochemical staining to detect expression of PSMA and CD31 was carried out on two consecutive 4-μm-thick paraffin sections. A ready-to-use monoclonal mouse anti-human PSMA antibody (DAKO, clone 3E6), and a ready-to-use monoclonal mouse anti-human CD31 antibody (DAKO, clone JC70A), were used.

Paraffin sections mounted on silanized slides were dried at 60 °C for 1 h, followed by antigen retrieval in TRS buffer (pH 9.0) for 20 min at 97 °C using the PT LINK Pre-Treatment Module for Tissue Specimens (DAKO). After washing in TBS buffer (pH 7.6), the slides were placed in the Autostainer LINK 48 (DAKO), and stained using a two-step polymer-based method. The EnVision™ Flex HRP High pH visualization kit (DAKO) was used as the detection system. Immunohistochemical staining using the Autostainer LINK 48 was performed according to the manufacturer’s protocol. After completion of immunostaining, the sections were dehydrated, embedded in mounting medium, and submitted for pathological evaluation.

**Figure 1:**
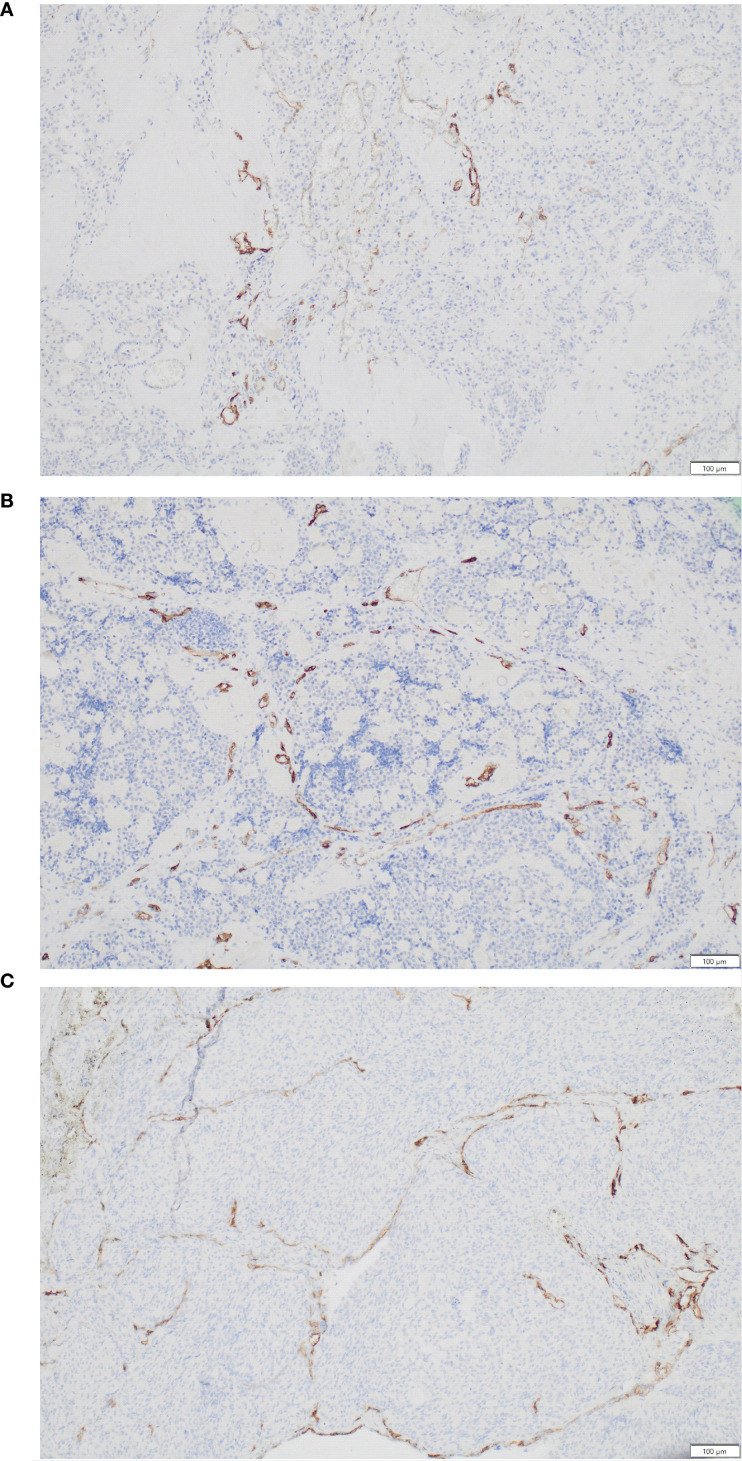
Different patterns of PSMA expression by medullary thyroid carcinoma. **(A)** PSMA expression by 10% of vessels (score 1). **(B)** PSMA expression by 15% of vessels (score 1). **(C)** PSMA expression by 55% of vessels (score 2).

Analysis of PSMA and CD31 immunostaining under a light microscope was conducted by an experienced pathologist. Evaluation of PSMA expression in the vascular endothelium of tumours was carried using the three-tiered scoring system proposed by Bychkov (23). Lack of PSMA expression or expression in <5% of microvessels was classified as negative (score 0). PSMA expression in ≥5% of microvessels was considered positive, with a score of 1 assigned to 5–50%, and a score of 2 to >50%. Examples of the PSMA immunostaining results are presented in **Figure 1**.

### 2.6 Laboratory tests

On the day of [^68^Ga]Ga-PSMA-11 PET/CT and [^18^F]FDG PET/CT imaging, serum levels of calcitonin and CEA were assessed using an electrochemiluminescence immunoassay and a Cobas e601 analyser (Roche, Mannheim, Germany). The measured ranges were 0.5–2000 pg/mL for calcitonin, and 0.2–1000 ng/mL for CEA.

### 2.7 Statistical analysis

Quantitative variables are expressed as the mean, median, lower and upper quartiles, minimum and maximum values, and standard deviation, while qualitative variables are expressed as numbers and percentages.

The normality of variable distribution was assessed using the Shapiro-Wilk test. Comparisons of serum calcitonin and CEA concentrations at the time of [^68^Ga]Ga-PSMA-11 PET/CT and [^18^F]FDG PET/CT, as well as semiquantitative PET parameters, were conducted using a paired samples Student’s t-test. When the assumptions for this test were not met, the Wilcoxon signed-rank test was applied. Correlation between PET-derived parameters (SUV_max_, TBR_Blood_, TBR_Liver_) and PSMA expression was assessed using Spearman’s rank correlation coefficient. Differences in the analysed variables, including calcitonin, CEA, calcitonin doubling time (DT), CEA DT, and PSMA expression, between groups with positive and negative [^68^Ga]Ga-PSMA-11 PET/CT results were assessed using the Mann-Whitney test. A significance level of p < 0.05 was adopted to indicate statistically significant differences or associations. Database management and statistical analyses were conducted using Statistica 9.1 software (StatSoft, Poland).

## 3 Results

### 3.1 Patient characteristics

Twelve patients (four male and eight female) with MTC who had undergone total thyroidectomy and presented with elevated serum calcitonin and/or CEA levels were enrolled in the study. Among these, three had previously-identified structural lesions: a superior mediastinal mass (patient no. 1), a metastatic cervical lymph node (patient no. 2), and five liver metastases (patient no. 3), giving a total of seven lesions. Nine patients showed no abnormalities on conventional imaging studies. The median age of the patients included in the study was 50 years (range, 39–65 years). Two patients had confirmed germline RET mutations (p.C620R, and a mutation in codon 791), and one patient had a somatic RET mutation (p.M918T). Patient characteristics and previous treatments are summarized in **Table 1**.

**Table 1:** Patient characteristics.

No.	Sex	Age	Year of diagnosis	TNM staging	Molecular pathology	Treatment received
1	F	64	2021	pT3aN1bR2	Sporadic, somatic RET mutation p.M918T	TT+CND+LND Superior mediastinal lymph node dissection Explorative thoracotomy
2	M	74	2018	pT1aN1b	Sporadic, somatic negative	TT+CND LND LND
3	M	42	2011	pT3NxM1	Sporadic, somatic unknown	TT+CND Liver metastasectomy [^131^I]I MIBG therapy, 27.75 GBq
4	F	33	2011	pT3N1b	Sporadic, somatic unknown	TT+CND+LND LND SRT for neck lymph node metastasis Octreotide LAR
5	F	38	2017	pT1b(m)N1b	Germline RET mutation p.C620R (MEN2A)	TT+CND+LND
6	F	44	2011	pT3N1b	Sporadic, somatic negative	TT+CND+LND LND
7	F	37	2019	pT1bN1b	Sporadic, somatic unknown	TT+CND LND EBRT
8	F	34	2012	pT1bN1a	Germline RET mutation in codon 791	TT+CND EBRT
9	M	63	2022	pT1b(m)N1b	Sporadic, somatic negative	TT+CND+LND EBRT
10	F	76	2018	pT1bN1b	Sporadic, somatic unknown	TT+CND+LND
11	F	40	2018	pT1bN1a	Sporadic, somatic unknown	TT+CND
12	M	65	2017	pT3NxM1R1	Sporadic, somatic unknown	TT+CND EBRT Excision of recurrence in the thyroidectomy bed Right upper lung lobectomy

CND, central neck dissection; EBRT, external beam radiation therapy; F, female; GBq, gigabecquerel; LAR, long-acting release; LND, lateral neck dissection; M, male; MEN2A, multiple endocrine neoplasia type 2A; MIBG, metaiodobenzylguanidine; RET, REarranged during Transfection proto-oncogene; SRT, stereotactic radiotherapy; TNM, tumour-node-metastasis; TT, total thyroidectomy.

**Figure 2:**
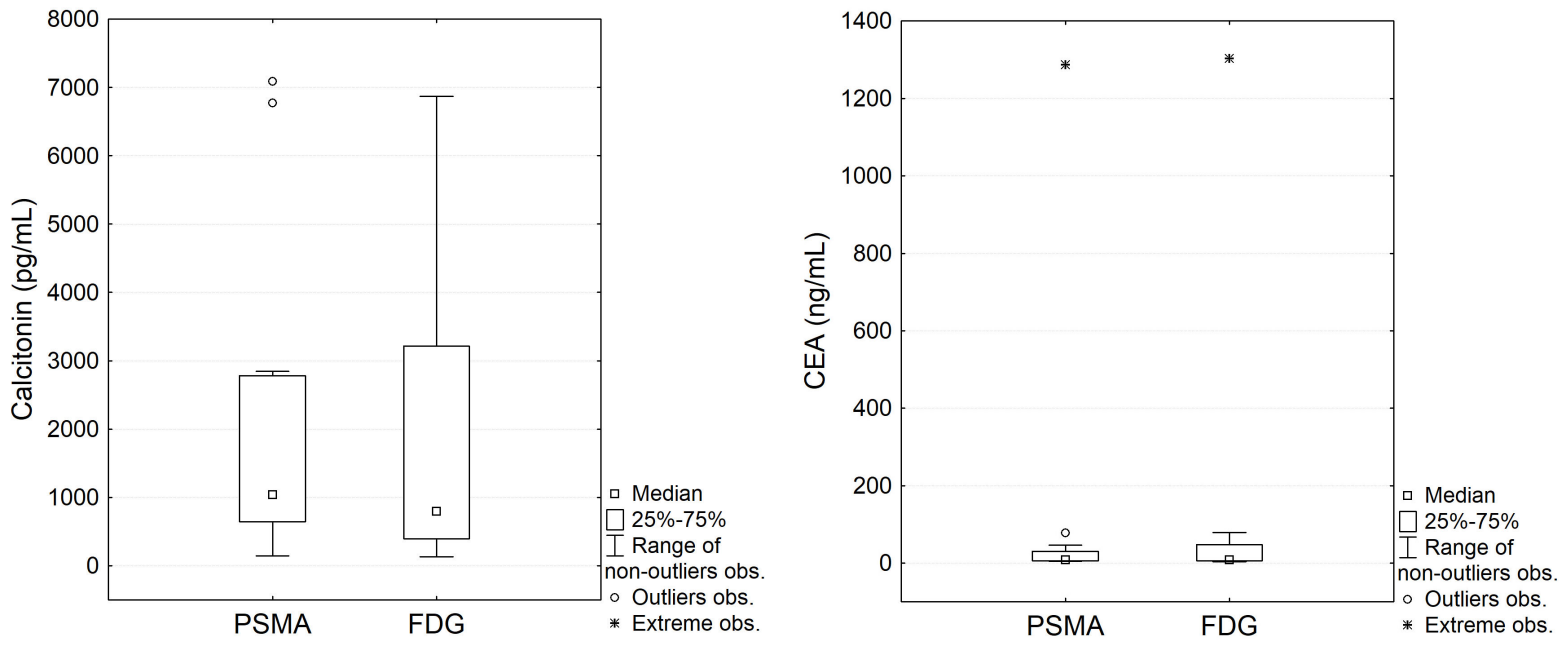
Comparison of serum calcitonin and CEA concentrations at the time of [^68^Ga]Ga-PSMA-11 PET/CT and [^18^F]FDG PET/CT.

All patients except for one (patient no. 2, who was referred for surgery after [^68^Ga]Ga-PSMA-11 PET/CT) underwent both [^68^Ga]Ga-PSMA-11 PET/CT and [^18^F]FDG PET/CT. The mean interval between PET scans was 109 days (median, 110; range, 82–129). The mean serum concentrations of calcitonin and CEA on the day of [^68^Ga]Ga-PSMA-11 PET/CT were 2125.03 pg/mL (median, 1038.40; range, 142.70–7088.00) and 123.33 ng/mL (median, 8.33; range, 4.24–1287.00), respectively, while on the day of [^18^F]FDG PET/CT they were 2108.55pg/mL (median, 798.90; range, 135.80–6875.00) and 135.55 ng/mL (median, 8.51; range, 3.64–1303.00), respectively. For the 11 patients who underwent both [^68^Ga]Ga-PSMA-11 and [^18^F]FDG PET/CT, there were no statistically significant differences in serum calcitonin levels (p=0.248) or CEA levels (p=0.131) between the two time points (**Table 2**, **Figure 2**). The mean calcitonin DT was 28.36 months, and the mean CEA DT was 33.27 months.

**Table 2:** Comparison of serum calcitonin and CEA concentrations at the time of [^68^Ga]Ga-PSMA-11 PET/CT and [^18^F]FDG PET/CT.

Parameter	PET/CT	N	M	Me	Min	Max	Q1	Q3	SD	Statistics
Ctn (pg/mL)	PSMA	12	2125.03	1038.40	142.70	7088.00	645.10	2781.50	2405.76	
	PSMA	11	2246.07	1115.00	142.70	7088.00	496.70	2849.00	2484.56	Z=1.156 **p=0.248**
	FDG	11	2108.55	798.90	135.80	6875.00	394.20	3217.00	2457.16	
CEA (ng/mL)	PSMA	12	123.33	8.33	4.24	1287.00	5.07	30.46	367.14	
	PSMA	11	133.88	9.23	4.24	1287.00	4.56	46.30	383.14	Z=1.511 **p=0.131**
	FDG	11	135.55	8.51	3.64	1303.00	5.80	47.68	387.92	

CEA, carcinoembryonic antigen; Ctn, calcitonin; FDG, [^18^F]FDG PET/CT; M, mean; Me, median; Mi, minimum; Ma, maximum; N, number of patients; Q1, lower quartile; PSMA, [^68^Ga]Ga-PSMA-11 PET/CT; Q3, upper quartile; SD, standard deviation; Z, Wilcoxon signed-rank test result; p, p-value.

Bolded – statistically significant values.

### 3.2 Imaging findings and analysis

**Figure 3:**
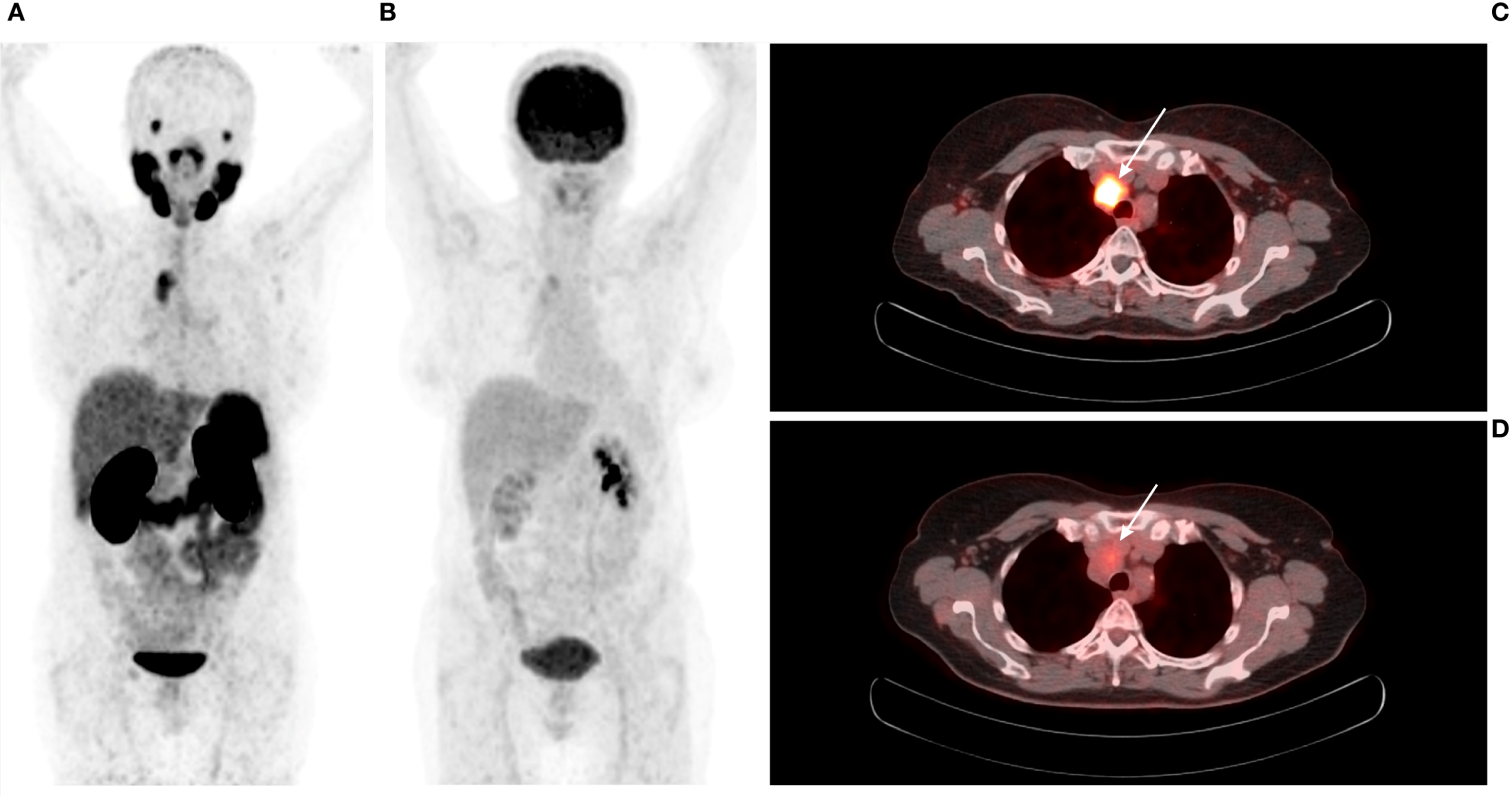
**(A)** 64-year-old female with medullary thyroid carcinoma and a superior mediastinal mass. **(A)** [^68^Ga]Ga-PSMA-11 PET MIP. **(B)** [^18^F]FDG PET MIP. **(C)** Transaxial view of the fused [^68^Ga]Ga-PSMA-11 PET/CT scan (white arrow). **(D)** Transaxial view of the fused [^18^F]FDG PET/CT scan (white arrow). The superior mediastinal mass is more avid on [^68^Ga]Ga-PSMA-11 PET (SUV_max_ 7.98; TBR_Blood_ 7.60) than on [^18^F]FDG PET (SUV_max_ 3.81; TBR_Blood_ 1.85).

**Figure 4:**
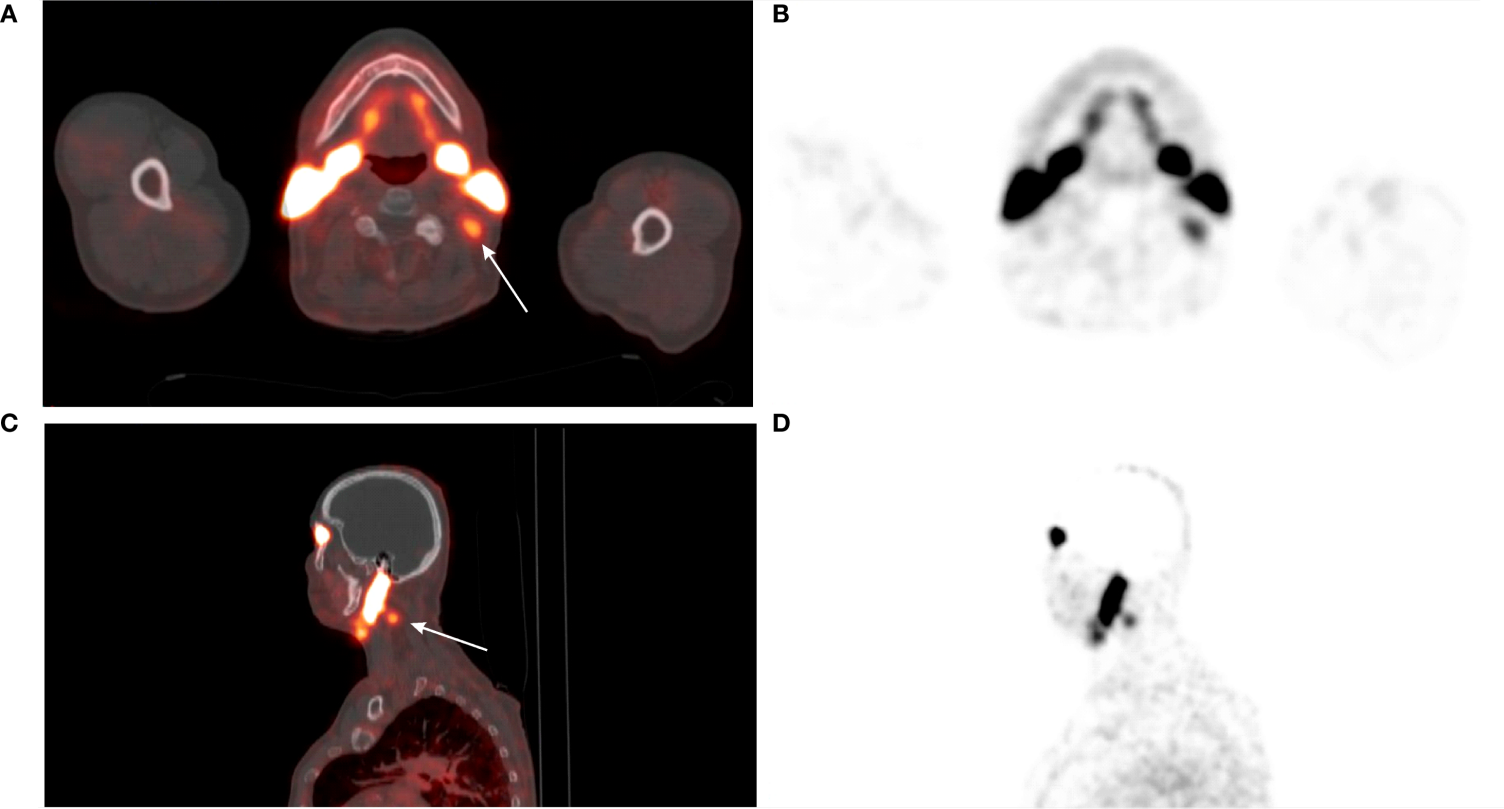
**(A)** 74-year-old male with medullary thyroid carcinoma and cervical metastatic lymph nodes. Transaxial **(A, B)** and sagittal **(C, D)** views of the fused [^68^Ga]Ga-PSMA-11 PET/CT and PET scans (white arrows). The metastatic lymph node in the left IIb neck compartment demonstrates abnormal [^68^Ga]Ga-PSMA-11 uptake (SUV_max_ 3.49; TBR_Blood_ 3.79). The patient underwent left cervical lymph node dissection. Histopathological examination confirmed metastasis of medullary thyroid carcinoma.

**Figure 5:**
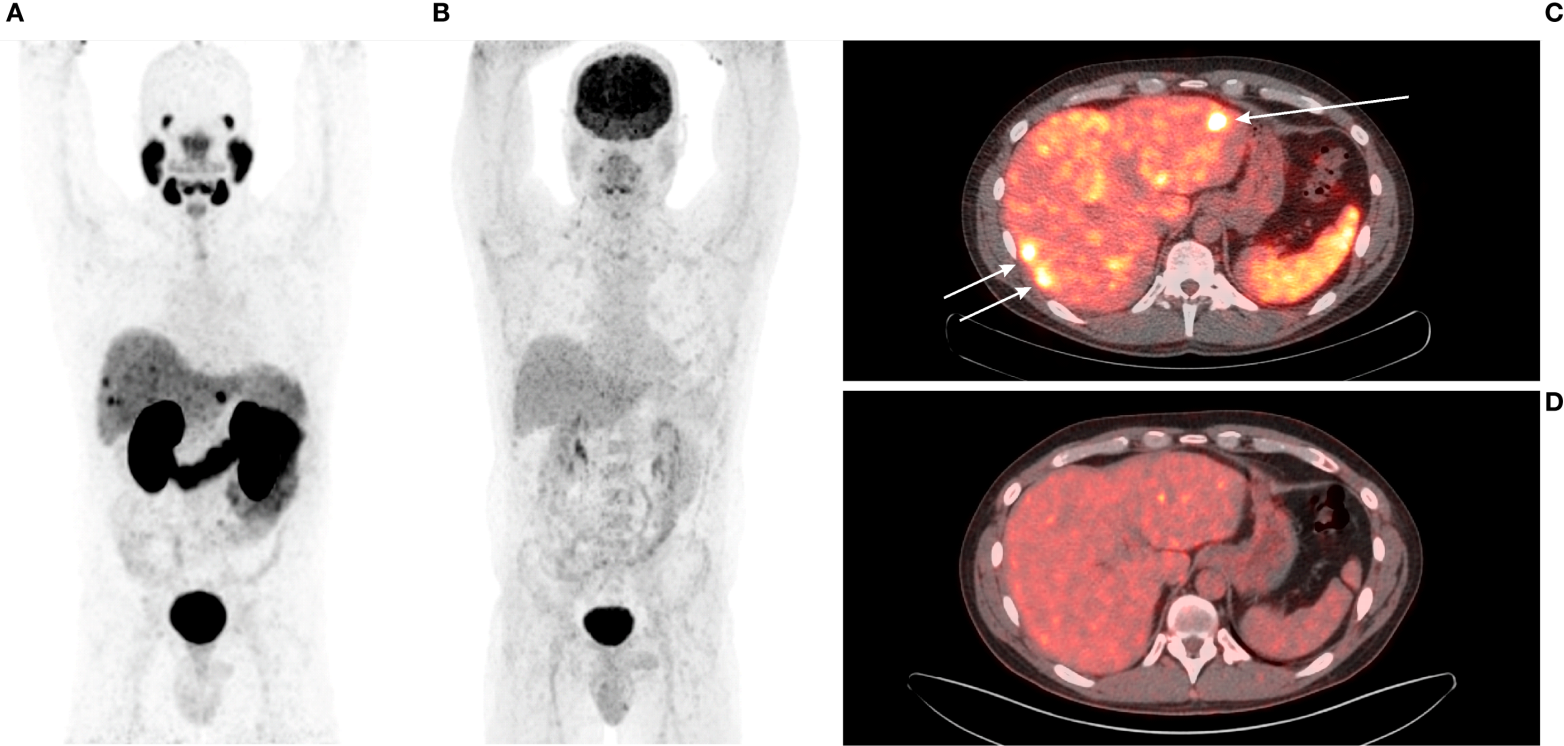
**(A)** 42-year-old male with medullary thyroid carcinoma and liver metastases. **(A)** [^68^Ga]Ga-PSMA-11 PET MIP. **(B)** [^18^F]FDG PET MIP. **(C)** Transaxial view of the fused [^68^Ga]Ga-PSMA-11 PET/CT scan (white arrows). **(D)** Transaxial view of the fused [^18^F]FDG PET/CT scan. Liver metastases are avid on [^68^Ga]Ga-PSMA-11 PET (mean SUV_max_ 9.25; mean TBR_Liver_ 2.82) and not avid on [^18^F]FDG PET (mean SUV_max_ 3.85; mean TBR_Liver_ 1.61). All metastases were undetectable on non-contrast CT (not shown in the Figure).

**Figure 6:**
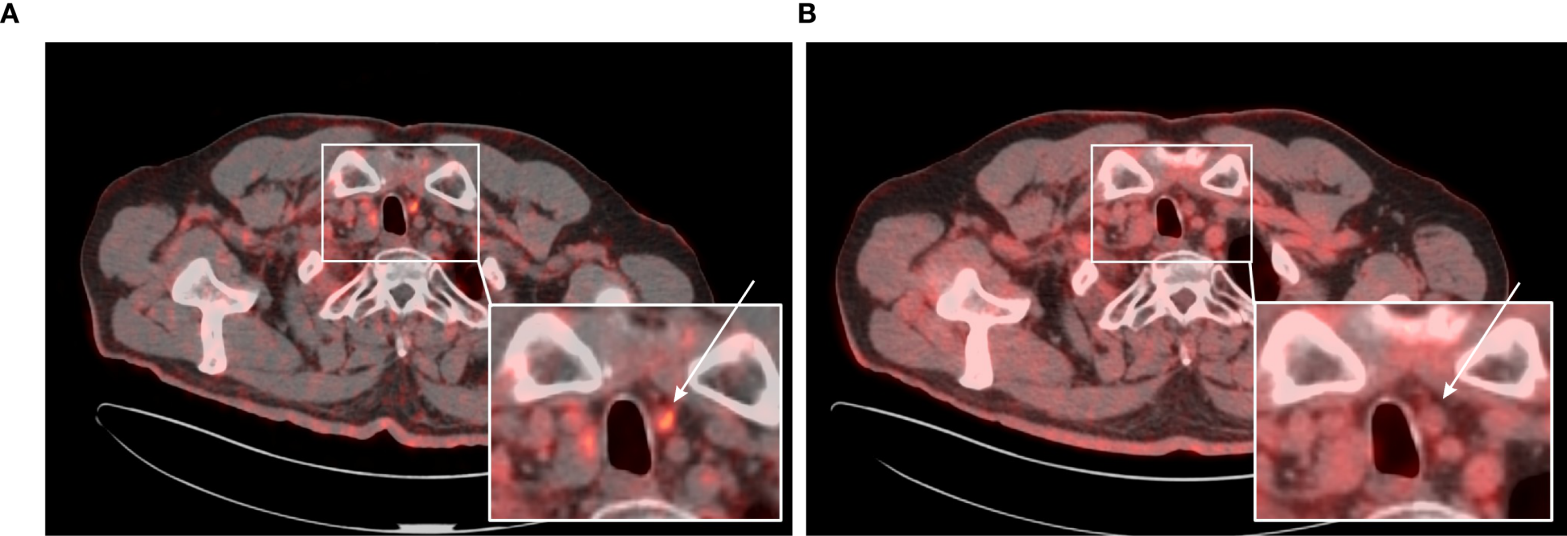
**(A)** 65-year-old female with medullary thyroid carcinoma and recurrence in thyroidectomy bed. **(A)** Transaxial view of the fused [^68^Ga]Ga-PSMA-11 PET/CT scan. **(B)** Transaxial view of the fused [^18^F]FDG PET/CT scan. In the thyroid bed on the left side, a spherical soft tissue lesion measuring 9 × 8 mm demonstrates increased [^68^Ga]Ga-PSMA-11 uptake (SUV_max_ 2.74; TBR_Blood_ 3.19) (white arrow). [^18^F]FDG PET/CT performed approximately 3 months later revealed that the same lesion, now measuring 11 × 10 mm, showed no evidence of increased [^18^F]FDG uptake (SUV_max_ 1.89; TBR_Blood_ 0.82) (white arrow). Fine-needle aspiration biopsy confirmed the lesion as a metastasis of medullary thyroid carcinoma.

[^68^Ga]Ga-PSMA-11 PET/CT was positive in 5/12 patients – 3/3 patients with previously-identified structural lesions (a superior mediastinal mass, a metastatic cervical lymph node, and liver metastases; **Figures 3**–**5**) and 2/9 patients without previously known structural lesions. In both of these cases, [^68^Ga]Ga-PSMA-11 PET/CT revealed small lesions in the thyroidectomy bed, which could correspond to local recurrence or lymph nodes (one shown in **Figure 6**). The mean SUV_max_, TBR_Blood_ and TBR_Liver_ values for detected lesions were 7.08 (range 2.74–12.37), 6.08 (range 3.19–9.82), and 2.23 (range 0.90–3.77), respectively.

[^18^F]FDG PET/CT was positive for 1/11 patients, demonstrating a previously-identified superior mediastinal mass (SUV_max_, 3.81; TBR_Blood_, 1.85; TBR_Liver_, 1.58), which was also positive on [^68^Ga]Ga-PSMA-11 PET/CT.

A summary of the [^68^Ga]Ga-PSMA-11 PET/CT and [^18^F]FDG PET/CT results, along with serum calcitonin and CEA measurements, is presented in **Table 3**.

**Table 3:** [^68^Ga]Ga-PSMA-11 PET/CT and [^18^F]FDG PET/CT results and laboratory findings.

Patient no.	Known lesions	Ctn DT (months)	CEA DT (months)	[^68^Ga]PSMA-11 PET/CT			[^18^F]FDG PET/CT			Interval between scans (days)
				Ctn (pg/mL)	CEA (ng/mL)	Result	Ctn (pg/mL)	CEA (ng/mL)	Result	
1	Yes	26.35	43.19	2849	46.30	Pos.	3217	47.68	Pos.	105
2	Yes	10.24	16.33	793.5	7.24	Pos.	N/A	N/A	N/A	N/A
3	Yes	47.35	55.57	2714	1287	Pos.	2432	1303	Neg.	129
4	No	46.33	52.96	6776	77.96	Neg.	6875	79.01	Neg.	111
5	No	15.42	33.00	961.8	11.44	Neg.	1227	12.48	Neg.	110
6	No	38.12	-123.87	820.6	4.24	Neg.	798.9	3.64	Neg.	121
7	No	15.56	32.69	7088	4.56	Neg.	6511	5.8	Neg.	125
8	No	20.01	49.40	496.7	5.58	Neg.	394.2	6.38	Neg.	95
9	No	20.30	34.45	1115	14.61	Neg.	698.4	13.65	Neg.	102
10	No	8.63	66.39	1564	9.23	Neg.	721.7	8.51	Neg.	120
11	No	40.39	82.85	179	7.43	Pos.	135.80	6.46	Neg.	99
12	No	51.64	56.25	142.7	4.37	Pos.	183	4.38	Neg.	82

CEA, carcinoembryonic antigen; Ctn, calcitonin; DT, doubling time; [^18^F]FDG, 2-[^18^F]fluoro-2-deoxy-D-glucose; [^68^Ga]Ga-PSMA-11, [^68^Ga]prostate-specific membrane antigen-11; N/A, not applicable; PET/CT, positron emission tomography/computed tomography.

Analysis of detection rates was conducted on 11 patients who underwent both [^68^Ga]Ga-PSMA-11 and [^18^F]FDG PET/CT imaging, excluding one patient who had a positive [^68^Ga]Ga-PSMA-11 PET/CT result but did not undergo [^18^F]FDG PET/CT (patient no. 2). Overall, the detection rates of [^68^Ga]Ga-PSMA-11 PET/CT were 4/11 (36.4%) for patient-based analysis and 8/8 (100%) for lesion-based analysis, whereas those for [^18^F]FDG PET/CT were 1/11 (9.1%) and 1/8 (12.5%), respectively. Both modalities yielded concordant results for 8/11 (72.7%) patients (one positive and seven negative on both scans) and discordant results for 3/11 (27.3%) patients, all of whom were positive on [^68^Ga]Ga-PSMA-11 PET/CT but negative on [^18^F]FDG PET/CT. [^68^Ga]Ga-PSMA-11 PET/CT altered the clinical management strategy for 1/12 patients (8.3%); this was patient no. 11, who was referred for surgical treatment.

SUV_max_, TBR_Blood_ and TBR_Liver_ were calculated for lesions shown as positive by at least one modality ([^68^Ga]Ga-PSMA-11 or [^18^F]FDG PET/CT; **Table 4**). A comparative analysis of SUV_max_, TBR_Blood_ and TBR_Liver_ was conducted between [^68^Ga]Ga-PSMA-11 PET/CT and [^18^F]FDG PET/CT in patients who underwent both scans, with the mean values from five liver lesions used for patient no. 3. For [^68^Ga]Ga-PSMA-11 PET/CT, the mean SUV_max_ was 5.80, with a mean TBR_Blood_ of 5.39 and a mean TBR_Liver_ of 1.93. The corresponding values for the same lesions on [^18^F]FDG PET/CT were 2.73, 1.56 and 1.13, respectively. There were statistically significant differences in TBR_Blood_ (p = 0.018) and TBR_Liver_ (p = 0.038) between the two imaging modalities, with higher values observed for [^68^Ga]Ga-PSMA-11 PET/CT in both cases (**Table 5**).

**Table 4:** Semiquantitative parameters of PET-positive lesions.

Patient no.	Known lesions	[^68^Ga]PSMA-11 PET/CT					[^18^F]FDG PET/CT				
		Result	Location	SUV_max_	TBR_Blood_	TBR_Liver_	Result	Location	SUV_max_	TBR_Blood_	TBR_Liver_
1	Yes	Pos.	Mediastinum	7.98	7.60	2.62	Pos.	Mediastinum	3.81	1.85	1.58
2	Yes	Pos.	Neck	3.49	3.79	1.08	N/A	N/A	N/A	N/A	N/A
3	Yes	Pos.	Liver	6.98 8.21 12.37 9.44 9.26	5.54 6.52 9.82 7.49 7.35	2.13 2.50 3.77 2.88 2.82	Neg.	Liver	3.54 3.44 4.19 3.92 4.14	2.49 2.42 2.95 2.76 2.92	1.48 1.44 1.75 1.64 1.73
11	No	Pos.	Thyroid bed	3.23	3.44	1.38	Neg.	Thyroid bed	1.38	0.87	0.64
12	No	Pos.	Thyroid bed	2.74	3.19	0.90	Neg.	Thyroid bed	1.89	0.82	0.71

[^18^F]FDG, 2-[^18^F]fluoro-2-deoxy-D-glucose; [^68^Ga]Ga-PSMA-11, [^68^Ga]prostate-specific membrane antigen-11; N/A, not applicable; Neg., negative; PET/CT, positron emission tomography/computed tomography; Pos., positive; SUV_max_, maximal standardized uptake value; TBR, tumour-to-background ratio.

**Table 5:** Comparison of SUV_max_, TBR_Blood_ and TBR_Liver_ values between [^68^Ga]Ga-PSMA-11 PET/CT and [^18^F]FDG PET/CT.

Parameter	PET/CT	M	Me	Min	Max	Q1	Q3	SD	Statistics
SUV_max_	PSMA	5.80	5.61	2.74	9.25	2.99	8.62	3.30	t=2.939 p=0.061
	FDG	2.73	2.85	1.38	3.85	1.64	3.83	1.28	
TBR_Blood_	PSMA	5.39	5.39	3.19	7.60	3.32	7.47	2.40	**t=4.677** **p=0.018**
	FDG	1.56	1.36	0.82	2.71	0.85	2.28	0.90	
TBR_Liver_	PSMA	1.93	2.00	0.90	2.82	1.14	2.72	0.94	**t=3.549** **p=0.038**
	FDG	1.13	1.15	0.64	1.61	0.68	1.59	0.53	

FDG, [^18^F]FDG PET/CT; M, mean; Me, median; Mi, minimum; Ma, maximum; PSMA, [^68^Ga]Ga-PSMA-11 PET/CT; Q1, lower quartile; Q3, upper quartile; SD, standard deviation; SUV_max_, maximum standardized uptake value; t, dependent samples t-test; TBR, tumour-to-background ratio; p, p-value

Bolded – statistically significant values.

### 3.3 Immunohistochemical staining results

Immunohistochemical staining for PSMA and CD31 was performed for 11/12 patients. CD31 expression was positive in all cases, confirming the presence of blood vessels in the analysed paraffin sections. PSMA expression was positive (score 1 or 2) in six out of 11 patients (55%) and negative (score 0) in the remaining five patients (45%). Positive expression was observed on the endothelial cells lining the tumour neovasculature, but not on tumour cells. All patients who were positive on [^68^Ga]Ga-PSMA-11 PET/CT were also positive for PSMA expression in immunohistochemical staining; the exception was patient no. 11, for whom immunohistochemistry could not be performed due to unavailability of histopathological samples. Two patients with positive PSMA immunostaining results were negative on [^68^Ga]Ga-PSMA-11 PET/CT (patients no. 5 and 9). The minimum level of PSMA expression observed on immunohistochemical staining required to yield a positive PET imaging result in this study was 7%. The results of immunostaining are summarized in **Table 6**.

**Table 6:** Immunostaining of PSMA and CD31.

Patient No.	1	2	3	4	5	6	7	8	9	10	11	12
CD31 expression (+/–)	+	+	+	+	+	+	+	+	+	+	NA	+
PSMA expression (%)	15	30	55	2	15	0	0	0	10	1	NA	7
PSMA score	1	1	2	0	1	0	0	0	1	0	NA	1

NA, not available; PSMA, prostate-specific membrane antigen

The correlation analysis between PET-derived parameters (SUV_max_, TBR_Blood_, TBR_Liver_) and PSMA expression was performed in four patients who were positive on [^68^Ga]Ga-PSMA-11 PET/CT and had available immunohistochemical staining. The correlation coefficients indicated a strong association of SUV_max_ (r = 0.800; p = 0.200) and TBR_Liver_ (r = 0.800; p = 0.200), and a moderate association of TBR_Blood_ (r = 0.400; p = 0.600) with PSMA expression; however, none of these results reached statistical significance.

### 3.4 Analysis of patients with positive and negative [^68^Ga]Ga-PSMA-11 PET/CT results

A comparative analysis of patients with positive and negative [^68^Ga]Ga-PSMA-11 PET/CT results, focusing on serum calcitonin levels, CEA levels, calcitonin and CEA DTs, as well as PSMA expression, was conducted (**Table 7**). PSMA expression was significantly higher in the group with positive [^68^Ga]Ga-PSMA-11 PET/CT results than in the group with negative [^68^Ga]Ga-PSMA-11 PET/CT results (p = 0.042). There were no statistically significant differences in other variables (calcitonin levels, CEA levels, calcitonin DT and CEA DT) between the two groups.

**Table 7:** Comparative analysis of patients with positive and negative [^68^Ga]Ga-PSMA-11 PET/CT results.

Variable	[^68^Ga]Ga-PSMA-11 PET/CT	N	M	Me	Min	Max	Q1	Q3	SD	Statistics
Ctn DT (months)	positive	5	35.19	40.39	10.24	51.64	26.35	47.35	16.93	U=9.0 p=0.202
	negative	7	23.48	20.01	8.63	46.33	15.42	38.12	13.58	
CEA DT (months)	positive	5	50.84	55.57	16.33	82.85	43.19	56.25	24.11	U=11.0 p=0.343
	negative	7	20.72	34.45	-123.87	66.39	32.69	52.96	64.98	
Ctn (pg/mL)	positive	5	1335.64	793.50	142.70	2849.00	179.00	2714.00	1345.82	U=11.0 p=0.343
	negative	7	2688.87	1115.00	496.70	7088.00	820.60	6776.00	2917.71	
CEA (ng/mL)	positive	5	270.47	7.43	4.37	1287.00	7.24	46.30	568.52	U=15.0 p=0.755
	negative	7	18.23	9.23	4.24	77.96	4.56	14.61	26.62	
PSMA expression (%)	positive	4	26.75	22.50	7.00	55.00	11.00	42.50	21.11	**U=2.5** **p=0.024**
	negative	7	4.00	1.00	0.00	15.00	0.00	10.00	6.03	

CEA, carcinoembryonic antigen; Ctn, calcitonin; DT, doubling time; M, mean; Me, median; Min –minimum; Ma, maximum; N, number of patients; Q1, lower quartile; Q3, upper quartile; SD, standard deviation; U, Mann-Whitney test; p, p-value; PET/CT, positron emission tomography/computed tomography; PSMA, prostate-specific membrane antigen.

Bolded – statistically significant values.

### 3.5 Follow-up findings

Follow-up data were available for all patients, with a mean duration of 678 days from study completion (median, 670; range, 486–912).

Patient no. 1, who presented with a superior mediastinal mass, underwent right-sided thoracotomy; however, resection was abandoned intraoperatively due to invasion of the superior vena cava and inability to achieve complete excision. The patient declined adjuvant radiotherapy. During the subsequent two years, serum calcitonin and CEA levels increased by 56% and 83%, respectively, while imaging demonstrated stable disease.

Patient no. 3 underwent core needle biopsy of a hepatic metastasis, which confirmed MTC. Over a 19-month period, calcitonin and CEA levels rose by 9% and 36%, respectively, with hepatic lesions remaining radiologically stable.

In patients no. 2, 11, and 12, cervical lesions detected on [^68^Ga]Ga-PSMA-11 PET/CT were cytologically confirmed as MTC. Two patients subsequently underwent surgery, whereas one remains under active surveillance, with stable lesions on ultrasonography and no biochemical progression.

In patient no. 8 (PET-negative), cervical recurrence was identified on ultrasonography 13 months after study completion, confirmed by biopsy and surgically resected. During this interval, calcitonin and CEA levels increased by 32% and 18%, respectively.

The remaining six PET-negative patients showed no pathological findings on follow-up imaging; however, in most cases, continued biochemical progression with rising calcitonin and CEA levels was observed.

## 4 Discussion

To date, published studies on the use of PSMA-targeted PET/CT in patients with MTC have been limited to case reports. Ciappuccini et al. presented the case of a 66-year-old man with prostate cancer who underwent [^68^Ga]Ga-PSMA-11 PET/CT and [^18^F]fluorocholine PET/CT imaging due to rising prostate-specific antigen levels to investigate biochemical recurrence. Both PET/CT scans showed incidental focal uptake in the right lobe of the thyroid. A neck ultrasound confirmed the presence of a 16-mm nodule. A FNAB was performed; however, the result was non-diagnostic (Bethesda I category). The patient underwent a right lobectomy, and histopathological examination confirmed the presence of MTC (35). Arora et al. reported two male patients who had undergone surgical treatment for MTC and presented with rising calcitonin and/or CEA levels. Both patients underwent [^68^Ga]Ga-PSMA PET/CT, which revealed locoregional recurrence with increased uptake of PSMA ligand (33, 34). Hasenauer et al. described the case of a 60-year-old man with MTC and hepatic, osseous, and lymph node metastases who underwent peptide receptor radionuclide therapy with [^177^Lu]Lu-DOTATOC. After two treatment cycles, a [^68^Ga]Ga-DOTATOC PET/CT scan was performed, revealing multiple nonavid lesions. Subsequently, [^18^F]F-PSMA-1007 PET/CT was conducted, which identified increased PSMA ligand uptake by both [^68^Ga]Ga-DOTATOC-avid and nonavid lesions (36). Another case was reported by Şahin et al., who described a 58-year-old patient with advanced MTC who underwent [^18^F]FDG, [^68^Ga]Ga-DOTATATE and [^68^Ga]Ga-PSMA PET/CT. Uptake of [^68^Ga]Ga-PSMA by metastatic lymph nodes in the left upper paraesophageal region and bone metastases was higher than that of [^18^F]FDG and [^68^Ga]Ga-DOTATATE. Metastatic lymph nodes in the right upper mediastinum showed mild [^68^Ga]Ga-PSMA uptake, whereas liver metastases exhibited no [^68^Ga]Ga-PSMA uptake. [^18^F]FDG and [^68^Ga]Ga-DOTATATE PET/CT demonstrated heterogeneous uptake of the tracer across metastatic lesions (37).

In the present study, we investigated the utility of [^68^Ga]Ga-PSMA-11 PET/CT for patients with MTC and elevated calcitonin levels indicative of persistent disease, recurrence, or distant metastases. To the best of our knowledge, this is the first study to compare the diagnostic performance of [^68^Ga]Ga-PSMA-11 PET/CT with that of [^18^F]FDG PET/CT in patients with MTC, and to correlate [^68^Ga]Ga-PSMA-11 PET/CT findings with PSMA immunohistochemical staining. In patients with previously-identified structural lesions, [^68^Ga]Ga-PSMA-11 PET/CT detected all seven known lesions in three patients, outperforming [^18^F]FDG PET/CT. PSMA-positive lesions included liver metastases, a superior mediastinal mass, and a metastatic cervical lymph node. In patients with hypercalcitoninemia and negative results on conventional imaging, [^68^Ga]Ga-PSMA-11 PET/CT detected lesions indicating recurrence in two out of nine patients (22%), both of whom were negative on [^18^F]FDG PET/CT. These were small recurrence foci in the neck, and in both cases FNAB confirmed MTC. Overall, the detection rates for [68Ga]Ga-PSMA-11 PET/CT were 8/8 (100%) for lesion-based analysis, and 4/11 (36.4%) for patient-based analysis, while rates for [^18^F]FDG PET/CT were 1/8 (12.5%) and 1/11 (9.1%), respectively. Both modalities yielded concordant results in 8/11 (72.7%) patients, and discordant results in the remaining 3/11 (27.3%) patients, all of whom were positive on [^68^Ga]Ga-PSMA-11 PET/CT but negative on [^18^F]FDG PET/CT.

The detection rates for [^18^F]FDG PET/CT observed in our study were lower than those reported previously. A meta-analysis by Treglia et al. (39) reveals that the pooled detection rate for [^18^F]FDG PET or PET/CT in a patient-based analysis of suspected recurrent or residual MTC was 59% (95% confidence interval: 54–63%); however, the studies included in this meta-analysis exhibited substantial heterogeneity with respect to detection rate estimates, ranging from 24%–88% (*I^2^* = 66%). Possible reasons for the discrepancy between our findings and the literature may include the high proportion of patients with hypercalcitoninemia, negative results from a wide range of conventional imaging modalities prior to PET/CT imaging, and exclusion of patients receiving anticancer treatment during the study. Additionally, the relatively long calcitonin and CEA doubling times (28.36 and 33.27 months, respectively) observed in our cohort may have contributed to the lower detection rates. It is well established that the detection rates of [^18^F]FDG PET and PET/CT are significantly higher for patients with a calcitonin DT of <12 months and a CEA DT of <24 months (39).

In 7/12 (58%) patients with hypercalcitoninemia, we detected no pathological lesions on either [^68^Ga]Ga-PSMA-11 or [^18^F]FDG PET/CT, nor on conventional imaging studies. This rate is higher than that reported by Lindsey et al. (20), who found that 35% of MTC patients present with a biochemical incomplete response to initial therapy, defined as detectable calcitonin or abnormal CEA in the absence of structural evidence of disease. A possible explanation is the presence of micrometastatic disease below the spatial resolution of PET, resulting in negative imaging despite biochemical evidence of disease. Negative [^68^Ga]Ga-PSMA-11 PET/CT may also result from lack of sufficient PSMA-expressing neovasculature; in our cohort, immunohistochemistry was negative in 5/11 patients, with most samples derived from primary tumors. Given the known intra-patient heterogeneity of PSMA expression, loss of expression in metastases cannot be excluded (40). By contrast, [^18^F]FDG uptake reflects glycolytic activity, which may be relatively low in indolent MTC. Finally, the absence of bone scintigraphy and whole-body MRI prior to study enrollment may have contributed to missed skeletal metastases (19, 41). Advanced MRI techniques, including whole-body MRI and dynamic contrast-enhanced liver MRI, may provide higher sensitivity than [^18^F]FDG PET/CT for detecting bone and liver metastases, supporting their complementary role in MTC follow-up (15).

In patients with biochemical incomplete response, conservative surveillance is recommended, with repeat measurement of serum markers every 6 to 12 months (14). Among patients with persistent hypercalcitoninemia, clinical or imaging-detected relapse occurs in approximately 40–45% of cases during prolonged follow up (42, 43); however, in some patients, even markedly elevated calcitonin levels may remain stable for many years, with a reported 10-year overall survival rate of 86% (42).

SUV_max_, TBR_Blood_ and TBR_Liver_ were calculated for lesions that were positive by at least one modality. The mean SUV_max_, TBR_Blood_ and TBR_Liver_ were 5.80, 5.39 and 1.93, respectively, for [^68^Ga]Ga-PSMA-11 PET/CT, while the corresponding values for [^18^F]FDG PET/CT were 2.73, 1.56 and 1.13. There was a statistically significant difference in TBR_Blood_ (p = 0.018) and TBR_Liver_ (p = 0.038) between the two imaging modalities, with [^68^Ga]Ga-PSMA-11 PET/CT demonstrating higher values in both cases. The TBR values observed in patients with MTC on [^68^Ga]Ga-PSMA-11 PET/CT were comparable with those reported in patients with biochemical recurrence of prostate cancer, with Heilinger et al. reporting values of 1.5 ± 2.5 for TBR_Liver_ and 5.1 ± 7.7 for TBR_Blood pool_ in [^68^Ga]Ga-PSMA-11 PET/CT (44). In the context of patient selection for PSMA-directed radioligand therapy for prostate cancer, the most important semiquantitative PET parameter is TBR_Liver_. In the VISION trial, eligibility required the presence of PSMA-positive tumour lesions, defined as those showing PSMA uptake above liver background activity on [^68^Ga]Ga-PSMA-11 PET/CT (45). Attempts are being made to apply the same eligibility criteria for radioligand therapy in patients with non-prostate cancers, including those with differentiated thyroid carcinoma and poorly differentiated thyroid carcinoma (46).

Immunohistochemical staining was performed for 11/12 (91.7%) patients. Expression of PSMA by endothelial cells lining the tumour neovasculature, but not tumour cells themselves, was observed. Positive expression of PSMA (defined as involvement of ≥5% of microvessels) was observed in 6/11 (55%) patients, while the remaining five (45%) were negative. All patients with positive [^68^Ga]Ga-PSMA-11 PET/CT results also exhibited expression of PSMA on immunohistochemical staining; the exception was one patient for whom immunohistochemistry could not be performed due to unavailability of histopathological samples. Most PSMA-positive patients had moderate expression levels (5–50%, score 1), whereas high expression (>50% of vessels involved, score 2) was found in only one patient. The minimal level of PSMA expression observed on immunohistochemical staining required to yield a positive [^68^Ga]Ga-PSMA-11 PET/CT result in our study was 7%. This value is consistent with the findings of Derlin et al., who reported that approximately 5% of PSMA-positive microvessels in thyroid adenoma were sufficient to allow significant accumulation of [^68^Ga]Ga-PSMA ligand on PET/CT imaging (47).

Previous studies show the proportion of MTC patients who were PSMA-positive on immunohistochemistry varied considerably, ranging from 33%–92% (22, 23, 38). In our study, the rate was 55%; however, in some cases, immunohistochemical staining of long-term preserved histopathological samples was performed (4/11 patients, specimens >10 years old), which may have reduced the sensitivity of the method, as membrane antigens such as PSMA are particularly prone to antigen decay in archival FFPE material (48). Additionally, the patients enrolled in our study constituted a selected cohort that showed an incomplete biochemical or structural response to treatment, classifying them as high-risk patients. This may have contributed to the lower proportion of immunohistochemistry-positive cases, as a higher number of PSMA-positive microvessels among MTC patients appears to be associated with a favourable prognosis (odds ratio 3.6; 95% confidence interval 1.0–12.8; p =0.05) (38).

Our study confirmed that PSMA expression by MTC is restricted to the endothelial cells of the tumour neovasculature. By contrast, PSMA expression in prostate cancer is localized to the membranes of tumour cells. This distinction carries important diagnostic implications. Expression of PSMA on the apical membrane of prostate cancer leads to intense PSMA accumulation on both immunohistochemistry and imaging studies (49), whereas in non-prostatic cancers, endothelial expression of PSMA may result in lower or more heterogeneous uptake of PSMA ligand (50). The correlation coefficients indicated a strong association of SUV_max_ and TBR_Liver_ and a moderate association of TBR_Blood_ with PSMA expression; however, the analysis included only four patients, and none of these associations reached statistical significance. Confirmation of these preliminary findings in a larger cohort would be valuable, considering that in some non-prostatic cancers there appears to be no consistent correlation between accumulation of PSMA ligands on PET/CT and PSMA expression in immunohistochemistry (51).

Published meta-analyses confirm that the diagnostic performance of [^18^F]FDG, [^18^F]FDOPA and [^68^Ga]Ga-SSA PET/CT in MTC patients improves when calcitonin and CEA levels are higher, as well as when calcitonin and CEA doubling times are shorter (39, 52, 53); however, our study did not confirm this association for [^68^Ga]Ga-PSMA-11 PET/CT. A possible explanation for this discrepancy could be the limited number of patients analysed in the current study. Nonetheless, we demonstrated that PSMA expression was significantly higher in the group with positive [^68^Ga]Ga-PSMA-11 PET/CT results than in those with negative PET results (p = 0.042). This suggests that evaluating PSMA expression by immunohistochemistry in already available surgical specimens, for example, from prior thyroidectomy, might provide additional information to help identify patients more likely to benefit from [^68^Ga]Ga-PSMA-11 PET/CT, although further research is needed to validate this approach.

This study has several limitations. The first and most significant is the small number of patients included, particularly those with previously-identified structural lesions. The small patient cohort was due to the rarity of MTC, and to the strict selection criteria applied. One of the exclusion criteria was the use of anticancer therapy between the[^68^Ga]Ga-PSMA-11 and [^18^F]FDG PET/CT scans. A substantial proportion of patients with metastatic MTC receive tyrosine kinase inhibitors, which did not allow them to meet the inclusion criteria. For the same reason, visceral and bone metastases are under-represented in our cohort, but are common findings in patients with advanced MTC (54). Therefore, the present results may not be directly applicable to patients with high-volume or rapidly progressive disease, and future studies should aim to include a broader spectrum of disease stages.

Furthermore, comparing [^68^Ga]Ga-PSMA-11 PET/CT with [^18^F]FDG PET/CT is suboptimal. According to meta-analyses, the pooled detection rates in per-patient analysis are 66% (95% confidence interval [CI]: 58%–74%) for [^18^F]FDOPA PET or PET/CT, 63.5% (95% CI: 49–77) for [^68^Ga]Ga-SSA PET or PET/CT, and 59% (95% CI: 54–63%) for [^18^F]FDG PET or PET/CT (39, 52, 53). Consistently, a network meta-analysis comparing PET radiopharmaceuticals demonstrated that [^18^F]FDOPA achieved the highest diagnostic performance for recurrent MTC in both patient- and lesion-based analyses, irrespective of serum calcitonin or CEA levels and calcitonin DT (55). However, recommendations regarding the use of different PET radiopharmaceuticals in MTC are inconsistent. The American Thyroid Association guidelines from 2015 state that neither [^18^F]FDG PET/CT nor [^18^F]FDOPA PET/CT is recommended to detect the presence of distant metastases in MTC (13), whereas the European Society for Medical Oncology guidelines from 2019 designate [^18^F]FDOPA as the preferred PET radiopharmaceutical in MTC, if available. [^18^F]FDG may be beneficial for evaluating advanced disease characterized by dedifferentiation and rapid progression. Additionally, [^68^Ga]Ga-SSA can be useful for assessing somatostatin receptor expression prior to radionuclide therapy, or when such treatment is being considered (15). By contrast, the National Comprehensive Cancer Network guidelines mention only [^18^F]FDG PET/CT and [^68^Ga]Ga-SSA as being useful, with no reference to [^18^F]FDOPA. The authors also emphasize that while many different imaging modalities can be used to search for residual or metastatic tumours, there is insufficient evidence that supports recommending any specific modality or combination of tests (14). However, in clinical practice, access to these radiopharmaceuticals remains the key limiting factor when deciding which one to use (56).

For an optimal assessment of the clinical utility of [^68^Ga]Ga-PSMA-11 PET/CT in MTC, head-to-head comparison with all three established tracers – [^18^F]FDG, [^68^Ga]Ga-SSA, and especially [^18^F]FDOPA as the most sensitive – would be most informative. However, [^18^F]FDOPA PET/CT was not available in our center, as in many others. Therefore, [^18^F]FDG PET/CT was selected as the comparator given its broad accessibility and routine clinical use.

In our study, [^68^Ga]Ga-PSMA-11 PET/CT yielded positive results predominantly in patients with relatively long calcitonin (mean, 35.19; median, 40.39; range, 10.24–51.64 months) and CEA DTs (mean, 50.84; median, 55.57; range, 16.33–82.85 months). In light of current recommendations, [^68^Ga]Ga-PSMA-11 PET/CT may represent a potential option when [^18^F]FDOPA PET/CT is unavailable or negative, especially in patients with moderate calcitonin and CEA DTs who are not candidates for SSA-based radionuclide therapy. Further studies, especially direct comparisons with [^18^F]FDOPA and [^68^Ga]Ga-SSA PET/CT, are warranted to better define the potential diagnostic role of [^68^Ga]Ga-PSMA-11 PET/CT in this setting.

Additionally, it would be valuable to determine whether uptake of PSMA ligand in PET/CT is associated with prognosis, because Lodjewik et al. found that PSMA expression on immunohistochemistry correlates positively with progression-free survival and overall survival of MTC patients (38). This could support the potential use of [^68^Ga]Ga-PSMA-11 PET/CT as a prognostic tool for this patient group.

## 5 Conclusions

Despite its limitations, this is the first study to compare the diagnostic performance of [^68^Ga]Ga-PSMA-11 PET/CT with that of [^18^F]FDG PET/CT for MTC, as well as the first to correlate [^68^Ga]Ga-PSMA-11 PET/CT findings with PSMA expression on immunohistochemistry. The data suggest that in MTC, PSMA is expressed by the endothelial cells of the tumour neovasculature, not by the tumour cells. Endothelial expression of PSMA was observed in 55% of MTC patients with postoperative elevation of calcitonin and/or CEA, with the majority exhibiting moderate expression. The detection rate of [^68^Ga]Ga-PSMA-11 PET/CT was 100% for lesion-based analysis and 36.4% for patient-based analysis, whereas for [^18^F]FDG PET/CT, the detection rates were 12.5% and 9.1%, respectively. TBR_Blood_ and TBR_Liver_ were significantly higher for lesions detected by [^68^Ga]Ga-PSMA-11 PET/CT than for lesions detected by [^18^F]FDG PET/CT. In addition, [^68^Ga]Ga-PSMA-11 PET/CT altered clinical management in one case. Studies with larger patient cohorts are needed to confirm the clinical utility of [^68^Ga]Ga-PSMA-11 PET/CT in patients with MTC.

## Data Availability

The original contributions presented in the study are included in the article/supplementary material. Further inquiries can be directed to the corresponding author.
